# Galectin-3 orchestrates the histology of mesentery and protects liver during lupus-like syndrome induced by pristane

**DOI:** 10.1038/s41598-019-50564-8

**Published:** 2019-10-10

**Authors:** F. S. Lemos, J. X. Pereira, V. F. Carvalho, E. S. Bernardes, R. Chammas, T. M. Pereira, R. S. Carvalho, R. Luisetto, M. C. El-Cheikh, S. Calil-Elias, F. L. Oliveira

**Affiliations:** 1 Programa de Pós-graduação em Ciências Aplicadas a Produtos para Saúde (PPGCAPS), Faculdade de Farmácia, Universidade Federal Fluminense, Niterói, Rio de Janeiro Brazil; 2 Instituto de Patologia Tropical e Saúde Pública, Universidade Federal de Goiás, Goiânia, Brazil; 3 Laboratório de Inflamação, Fundação Oswaldo Cruz, Rio de Janeiro, Brazil, Instituto Nacional de Ciência e Tecnologia - Neuroimunomodulação (INCT-NIM), Rio de Janeiro, Brazil; 4 Centro de Radiofarmácia, Instituto de Pesquisas Energéticas e Nucleares (IPEN), São Paulo, SP Brazil; 5 Laboratório de Oncologia Experimental e Instituto do Câncer do Estado de São Paulo, Faculdade de Medicina, São Paulo, Brazil; 6 Laboratório de Alvos Moleculares, Departamento de Biotecnologia Farmacêutica, Faculdade de Farmácia, Universidade Federal do Rio de Janeiro, UFRJ, Rio de Janeiro, Brazil; 7 Department of Surgery, Oncology and Gastroenterology, University of Padova, Padova, Italy; 8 Instituto de Ciências Biomédicas, Universidade Federal do Rio de Janeiro, Rio de Janeiro, Brazil

**Keywords:** Cell biology, Inflammation

## Abstract

Galectin-3 (Gal-3) controls intercellular and cell-extracellular matrix interactions during immunological responses. In chronic inflammation, Gal-3 is associated with fibrotic events, regulates B cell differentiation and delays lupus progression. Gal-3 deficient mice (Lgals3^−/−^) have intense germinal center formation and atypical plasma cell generation correlated to high levels IgG, IgE, and IgA. Here, we used pristane (2,6,10,14-tetramethylpentadecane) to induce lupus-like syndrome in Lgals3^−/−^ and Lgals3^+/+^ BALB/c mice. Mesentery and peritoneal cells were monitored because promptly react to pristane injected in the peritoneal cavity. For the first time, mesenteric tissues have been associated to the pathogenesis of experimental lupus-like syndrome. In Lgals3^+/+^ pristane-induced mice, mesentery was hallmarked by intense fibrogranulomatous reaction restricted to submesothelial regions and organized niches containing macrophages and B lymphocytes and plasma cells. In contrast, Lgals3^−/−^ pristane-treated mice had diffuse mesenteric fibrosis affecting submesothelium and peripheral tissues, atypical M1/M2 macrophage polarization and significant DLL1^+^ cells expansion, suggesting possible involvement of Notch/Delta pathways in the disease. Early inflammatory reaction to pristane was characterized by significant disturbances on monocyte recruitment, macrophage differentiation and dendritic cell (DC) responses in the peritoneal cavity of pristane-induced Lgals3^−/−^ mice. A correlative analysis showed that mesenteric damages in the absence of Gal-3 were directly associated with severe portal inflammation and hepatitis. In conclusion, it has suggested that Gal-3 orchestrates histological organization in the mesentery and prevents lupoid hepatitis in experimental lupus-like syndrome by controlling macrophage polarization, Notch signaling pathways and DC differentiation in mesenteric structures.

## Introduction

Galectin-3 (Gal-3) is a β-galactoside binding protein which exerts pleiotropic biological functions involving intercellular and cell-extracellular matrix interactions, such as cellular differentiation, angiogenesesis and metastasis^1^. This protein is widely distributed throughout several tissues, in both intracellular and extracellular compartments. Furthermore, its secretion is non-classical pathway^2^. The advent of Gal-3 deficient mice (Lgals3^−/−^ mice) revealed modulatory roles on granulocyte and macrophage functions during acute inflammatory responses^3,4^. In chronic responses, Gal-3 is associated with fibrotic events in several tissues, including liver, kidney and mesentery^5–7^.

Lgals3^−/−^ mice show significant histological and physiological changes in B cell niches in the bone marrow, spleen, mesenteric lymph nodes and mesentery of mice chronically-infected with *Schistosoma mansoni* and *Trypanosoma cruzi*^8–12^. These mice have an intense plasmacytogenesis in lymphoid organs and high levels of serum immunoglobulins, including IgG, IgE, and IgA. The exacerbated number of plasma cells in Lgals3^−/−^ mice suggested the involvement of this lectin in the pathogenesis of autoimmune responses^13–16^. Recently, Beccaria and colleagues revealed that Lgals3^−/−^ developed a lupus-like disease hallmarked by intense germinal center B cells, Ig-secreting cells, and IFN-γ signature^17^.

Pristane (2,6,10,14-tetramethylpentadecane) induces polyclonal activation on B lymphocytes associated with symptoms of systemic lupus erythematosus (SLE), such as autoantibody secretion and glomerulonephritis^18,19^. SLE is hallmarked by autoantibody-producing B lymphocytes^20^ and serum autoantibodies from atypical plasma cells detected in humans and experimental animals^21^. Moreover, pristane intraperitoneally injected favors leukocyte recruitment and oil granuloma formation (lipogranulomas) in the mesentery^18^. Pristane-induced lupus-like experimental model has been used to understand pathogenesis and cellular mechanisms of SLE. In this model, the INF signature and autoimmune manifestations affecting multiple organs are significantly associated with dendritic cells (DCs) that promptly respond to pristane injection in the peritoneal cavity^22–24^.

Here, we considered the mesentery in the context of lupus-like pristane-model. The recent inclusion of mesentery as organ^25^ reinforced our propose to investigate a possible participation of mesentery in the pathogenesis of experimental SLE induced by pristane. Mesenteric Gal-3 organizes lymphoid niches, submesothelial fibrosis and M1/M2 macrophage polarization by mechanisms that include Notch/Delta signaling pathways. These findings were significantly associated with liver steatosis. On the other hand, mesenteric injuries in Lgals3^−/−^ mice were correlated with intense inflammatory reaction in the hepatic portal zone and hepatitis. We convey that mesentery protects the liver by inhibiting experimental lupoid hepatitis.

## Material and Methods

### Mice

Eight-week old Lgals3^+/+^ and Lgals3^−/−^ BALB/c mice (female) were obtained from the colony bred at the Federal University of Rio de Janeiro, Brazil. Lgals3^−/−^ mice were generated and backcrossed to BALB/c mice as previously described^4^. All experimental methods involving mice were performed in accordance with guidelines and regulations were approved by Brazilian College of Animal Experimentation (CONCEA - Conselho Nacional de Controle de Experimentação Animal) and Animal Ethics Committee (CEUA, Comissão de Ética no Uso de Animais) of Federal University of Rio de Janeiro, Brazil (number DAHEICB071).

### Pristane model (Lupus-like model)

Female randomized Lgals3^+/+^ or Lgals3^−/−^ mice received a single intraperitoneal injection (i.p.) of 500 µL (16.7 mL/Kg/mouse) of pristane (2,6,10,14 tetrametyl-pentadecane; Sigma-Aldrich, USA). Lupus-like syndrome in mice is characterized after six months in contact with pristane^26^. Here, we analyzed two endpoints: 6 months to study experimental SLE and 18 hours after the i.p. injection of pristane to evaluate early events associated with inflammatory response to pristane. Non-injected mice were used as controls.

### Histological analysis of mesentery

On day 180 after pristane injection, the mesentery of each Lgals3^+/+^ or Lgals3^−/−^ mouse was collected after exposure of the peritoneal cavity and immediately fixated in 10% buffered formalin pH 7.4 for 24 h at 4 °C. Samples were dehydrated in growing concentrations of ethanol (70–100%) for 30 min each and washed 2 times in xylene for 15 min. Then, mesenteries were individually embedded in paraffin and tissues were sectioned into 3 μm thick slices and stained with hematoxylin and eosin. Picrosirius red stain was utilized to identify fibrosis. Bright-field pictures were acquired using an Evolution MP 5.0 RTV Color camera (Media Cybernetics, Canada).

### Immunohistochemistry

Paraffin-embedded sections of 3 μm were dewaxed using xylene, hydrated in a graded ethanol and washed with distilled water. After inhibition of endogenous peroxidase, sections were treated for 1 h with BSA 8% and diluted in 0.002% PBS-Tween 20. Tissues were incubated with primary antibodies (1 h), washed twice with 0.002% PBS-Tween 20, incubated with secondary antibody (1 h), and newly washed to follow addition of streptavidin−peroxidase for 1 h (Sigma-Aldrich, USA). Reactions were revealed by diaminobenzidine as the chromogen and samples were counterstained with Harris’ hematoxylin. Primary antibodies: polyclonal anti-galectin-3 (clone M3/38), Delta-like-1 and Delta-like-4 (Santa Cruz Biotechnology, USA), and monoclonal B220, CD138, Arg-1, iNos (BD Bioscience, USA).

### Peritoneal cell suspensions

Peritoneal cells were harvested on cold phosphate buffered saline (PBS), pH 7.2, with 3% fetal bovine serum (LGC, Brazil) in 1 mL final volume. Cells were quantified by hemocytometer and samples were adjusted to 1 × 10^6^ cells/mL or 2 × 10^4^ cells/mL for flow cytometry and cytosmears analysis, respectively.

### Flow cytometry

Fc receptors of peritoneal cells were blocked (Fc blocker antibody, clone 2.4G2) for 10 min. The following monoclonal antibodies were used for membrane staining: anti-Mac-1/CD11b, B220 (Fluorescein isothiocyanate, FITC); anti-Mac-3, CD62L, CD23, CD49e, B220 (phycoerithrin, PE); anti-Gr1 (phycoerithrin Cychrome, PECy5.5); anti-CD11c (allophycocyanin, APC). Samples were acquired in FACScalibur (BD Bioscience, USA) using software Cell Quest and analyzed in both Cell Quest and WinMDI 2.9. All experimental procedures with flow cytometry were performed using 5 mice per group in 3 independent experiments.

### Peritoneal cytosmears

Resident peritoneal floating cells obtained from Lgals3^+/+^ and Lgals3^−/−^ mice were harvested under sterile conditions as described previously. Cytosmears were performed in 300 μl PBS, centrifuged at 36 *g* for 3 min (Thermo Scientific Cytospin 4 Cytocentrifuge, Mass., USA), fixed in absolute methanol for 24 h and stained with May-Grünwald-Giemsa^11^. Morphological analysis was performed by using high-power microscopy (Zeiss-Axioplan, Germany). The images were acquired by bright field microscopy using an Evolution MP 5.0 RTV-Color camera (Media Cybernetics, Canada).

### Immunocytochemistry

Peritoneal cells were centrifuged by cytosmear on glass slides coated with poly-L-lysine and fixed in methanol for 24 h at room temperature. After inhibition of endogenous peroxidase, cytosmears were incubated for 1 h with PBS containing 5% BSA, 4% skim milk, 0.1% Triton x-100 (Sigma Aldrich, USA), 0.05% Tween-20, and 10% normal goat serum and incubation with purified rat IgG anti-F4/80 (BD Biosciences, USA) for 4 h at 4 °C in a humid chamber. Antibodies were detected with a biotinylated anti-rat IgG (BA-4001, Vector Laboratories, Burlingame, CA, USA) and developed with avidin-peroxidase (Sigma Aldrich, USA), using diaminobenzidine as chromogen. Slides were counterstained with Harris’hematoxylin. Bright-field pictures were acquired using an Evolution MP 5.0 RTV Color camera (Media Cybernetics, Canada).

### Hematological parameters

Blood was obtained from cardiac puncture and stored in contact with EDTA solution. To red blood cell (RBC) count, samples were diluted 1:200 dilution of blood in Gower’s solution (20 µl of blood + 3980 µl of Gower’s solution). After continuous mixing for 3 minutes using mechanical shaker, 10 µl were used in the hemocytometer to count erythrocytes in light microscopy with (40×) objective lens passing through all the squares in the chamber. To count white blood cells (WBCs or leukocytes), the four large squares in the corner of hemocytometer were used. Previously, RBCs were lysed in ammonium-chloride-potassium (ACK) solution and samples were diluted 1:20 (100 µl of blood + 1900 µl of ACK’s solution). Approximately 10 µl of diluted solution was used in the chamber and WBCs were counted on the light microscope using (10×) objective lens to quantify all cells distributed by the four corner squares. To platelets count, blood with EDTA was diluted with 1% ammonium oxalate (20 µl of blood + 1980 µl of 1% ammonium oxalate) and samples were continuously mixed for 5 minutes The hemocytometer was filled by 10 µl of diluted solution and platelets were counted in the squares after 15 minutes using (40×)×) objective lens. To each counting, the cells in contact with top and left lines were quantified but cells touching bottom and right lines were ignored. The calculation of the RBCs, WBCs and platelets was defined by Total count (cells/L) = (cells counted × dilution × 10^6^)/volume. Volume is specific to squares used to count each cell type. Data were adjusted to cells/mm^3^ of blood.

### Mesentery dissociation (AnnexinV and PI test)

Mesentery was removed and enzymatically dissociated in 20–30 mL of culture medium (alpha-MEM, pH 7.4) containing collagenase 1 A, trypsin IX and papain 2x crystallized (Sigma-Aldrich, USA) at 37 °C in 5% CO_2_ atmosphere for 2 hours with gentle agitation. At the end of the incubation, the cells recovered by centrifugation (5 min, 150 g). Each pellet was submitted to annexin V-FITC and propidium iodide (PI) staining and flow cytometry analysis. Living cells have double-negative Annexin-V^−^PI^−^ phenotype while dead cells are positive to Annexin-V and/or PI. Results were representative of three independent experiments.

### Mesentery dissociation (Real-Time PCR)

Mesentery was removed and enzymatically dissociated as described above. To RT-PCR, each pellet was submitted to RNA extraction using the TRIzol reagent (Invitrogen Life Technologies, USA), following the manufacturer’s instructions.

### Real Time – PCR

The mRNA levels of Histocompatibility 2 class II antigen A beta 1 (*H2-Ab1*), Tumor necrosis factor (*Tnf*), Macrophage galactose N-acetyl-galactosamine-specific lectin 2 (*Mgl2*), Transforming growth factor, beta 1 (*Tgfb1*) and Glyceraldehyde-3-phosphate dehydrogenase (*Gapdh*) were quantified using Hot FirePol Evagreen^®^ qPCR mix (Solys Biodyne) and specific primers listed below. All primers were purchased from Thermo Fisher Scientific. Total RNA was isolated from mesenteric samples using Trizol^®^ reagent (Invitrogen) according to the manufacturer’s instructions and quantified using Nanodrop^®^ spectrophotometer. Three micrograms of total RNA were used for cDNA synthesis, using RevertAid cDNA synthesis kit (Thermo Fisher Scientific). Each sample was analyzed in triplicate using a LineGene 9600 thermocycler (Bioer Technology). The relative quantification of target genes was performed by ΔΔCt method using *Gapdh* as endogenous control.

**Table:** 

Gene	Forward primer	Reverse primer
*MhcII*	5′-TTTGCTTTCTGAAGGGGGCA-3′	5′-TCGCCCATGAACTGGTACAC-3′
*Tnf*	5′-TACTGAACTTCGGGGTGATCGGTCC-3′	5′-CAGCCTTGTCCCTTGAAGAGAACC-3′
*Tgfb1*	5′-TACCATGCCAACTTCTGTCTGGGA-3´	5′-ATGTTGGACAACTGCTCCACC-3´
*Mgl2*	5′-TTCAAGAATTGGAGGCCACT-3′	5′-CAGACATCGTCATTCCAACG-3′
*Gapdh*	5′-AGGTCGGTGTGAACGGATTTG-3´	5´-TGTAGACCATGTAGTTGAGGTCA-3´

### Statistical analysis

The statistical tests were accomplished using Tukey’s multiple comparison test (t-test), and significance threshold was fixed for α = 0.05. Therefore, P values ≤ 0.05 were considered statistical differences. Each experiment was performed using 3–5 mice per group in three independent assays.

## Results

### Gal-3 organizes fibrotic mesenteric niches in response to pristane

**Figure 1:**
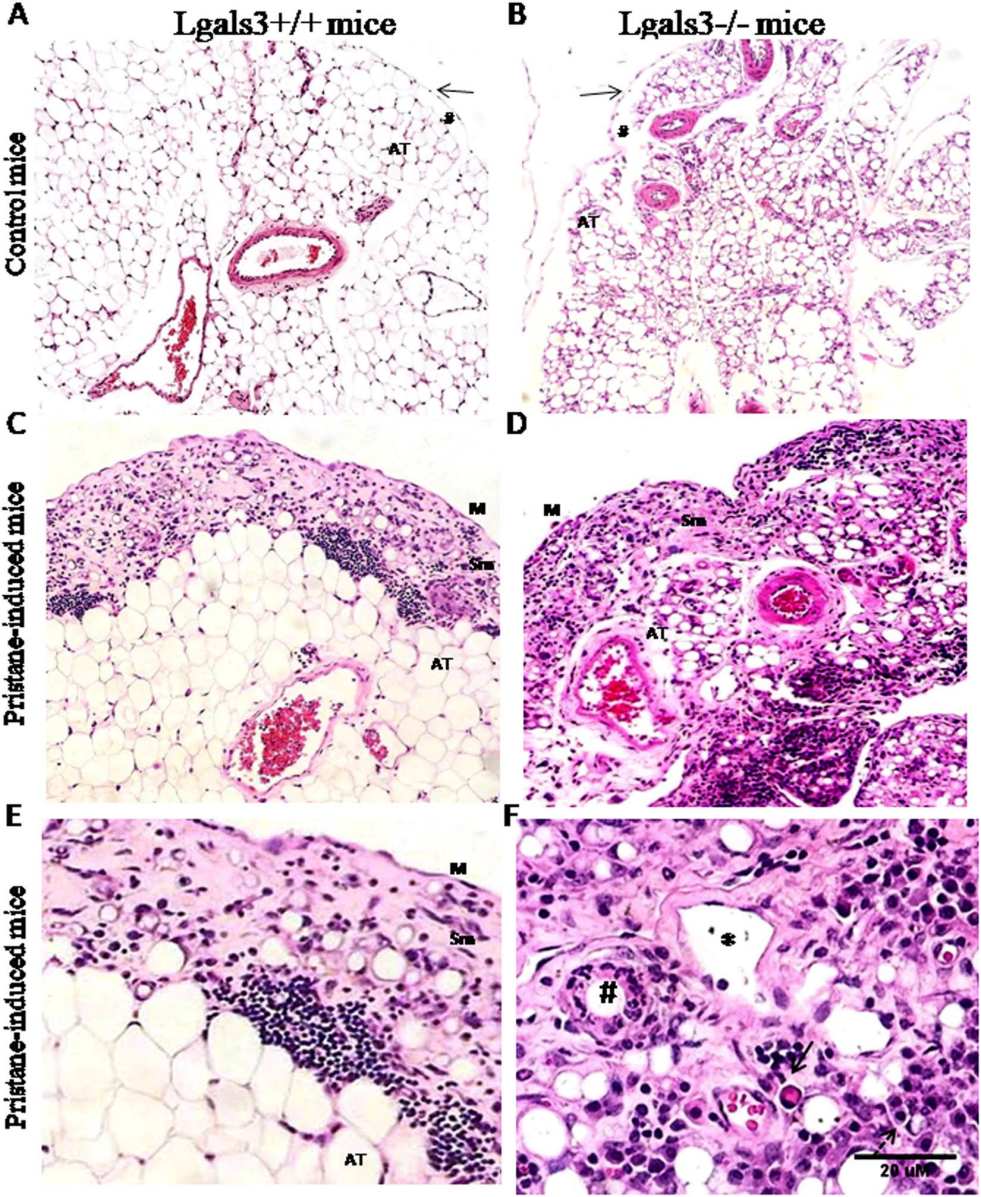
Photomicrographs of mesentery on pristane-induced mice. Representative images of mesenteric tissues obtained from control Lgals3^+/+^ (**A**) and Lgals3^−/−^ mice (**B**). Pristane induced submesothelial inflammatory reaction in Lgals3^+/+^ mice (**C**) and diffuse in the absence of galectin-3 (**D**). M: mesothelium, Sm: submesothelium, and AT: adipose tissue. (**E**) Milky spot-like structures were observed in the submesothelial tissue of pristane-induced Lgals3^+/+^ mice. In Lgals3^−/−^, clusters of lymphocytes were disorganized showing atypical plasma cells (bold arrow), lymphatic dilation (*), myeloid reaction to lipid-stored area (#) and LE cells-like (**F**). Data are representative of three independent experiments. Magnification: A-D (100×) and E-F (500×).

In control condition, the mesentery of Lgals3^+/+^ mice showed unilocular adipose tissue surrounded by a tiny submesothelial layer and mesothelial tissues (Fig. 1A). In non-stimulated Lgals3^−/−^ mice, all these structures were found in the mesentery. However, the adipose tissue was typically multilocular (Fig. 1B). Pristane induces a significant submesothelial reaction 6 months after i.p. injection characterized by extracellular matrix deposition and milky spots well defined by lymphocyte-clusters in Lgals3^+/+^ mice (Fig. 1C). On the other hand, the submesothelium was poorly identified in pristane-treated Lgals3^−/−^ mice due to abnormal distribution of extracellular matrix and leukocyte infiltrate (Fig. 1D). Mononuclear cells were predominantly found surrounding fat storing regions in submesothelial tissue of pristane-induced Lgals3^+/+^ mice (Fig. 1E). In contrast, granulocytes were frequently observed in similar lipidic structures in the mesentery of pristane-induced Lgals3^−/−^ mice. Atypical plasma cells, lymphangiectasia and LE-like cells were also observed in these mice knockout for Gal-3 (Fig. 1F).

**Figure 2:**
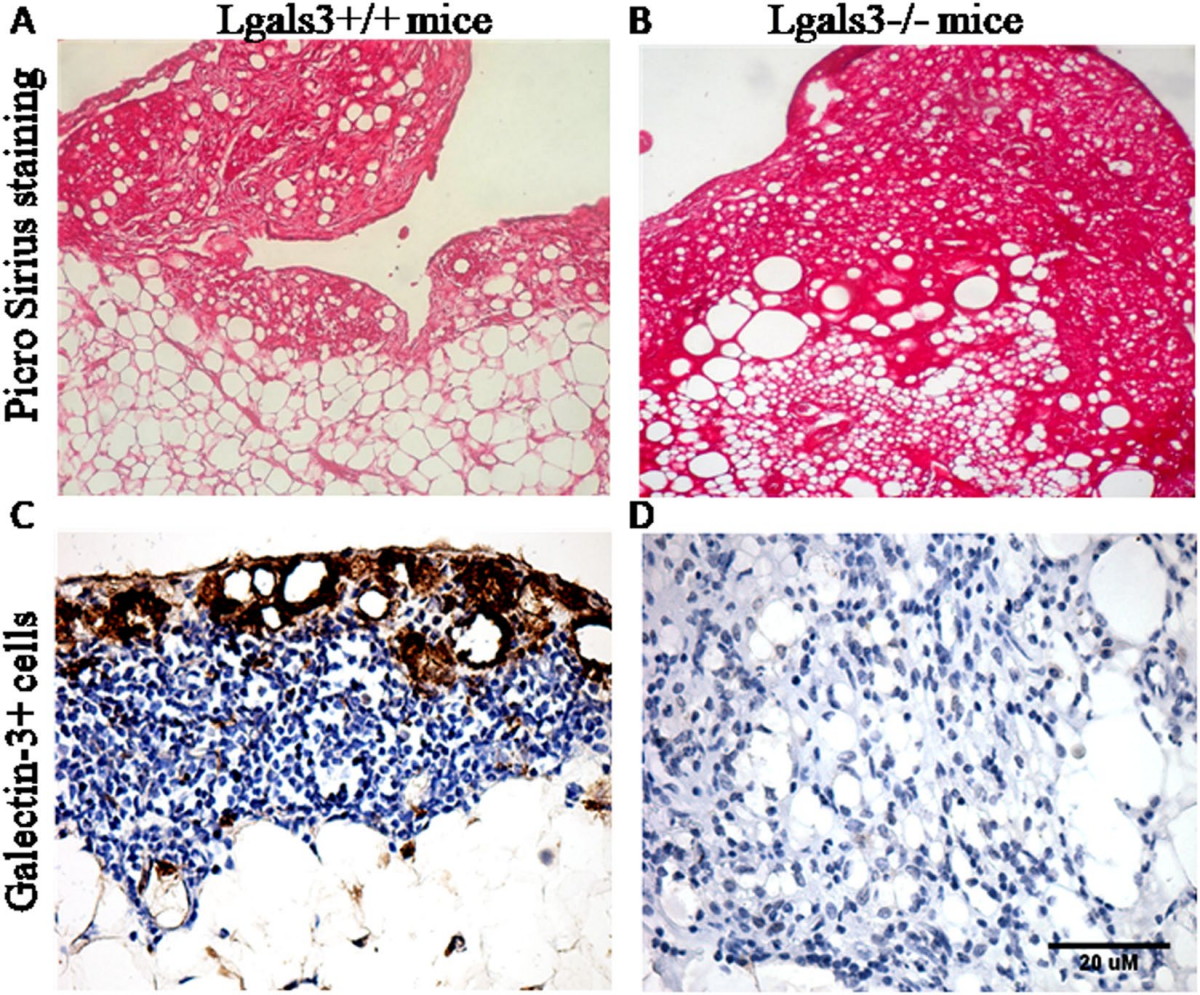
Photomicrographs of fibrotic mesentery and correlation with galectin-3 on pristane-induced mice. Pristane induced a well-defined submesothelial fibrosis in Lgals3^+/+^ mice (**A**). In Lgals3^−/−^ pristane-induced mice, fibrosis was severely dispersed throughout submesothelium and adipose tissue of mesentery (**B**). Staining: picrosirius red. By immunohistochemistry, Gal-3^+^ cells were preferentially found in the submsothelial regions in non-lymphoid regions (**C**). As expected, Gal-3^+^ cells were not observed in Lgals3^−/−^ pristane-induced mice (**D**). Data are representative of three independent experiments. Magnification: A-B (100×), C-D (200×).

The atypical extracellular matrix found in submesothelial regions in Lgals3^−/−^ mice stimulated with pristane led us to investigate collagen deposition and fibrotic reactions in the mesentery of these animals. Pristane induced a fibrosis restricted to submesothelium in the mesentery of Lgals3^+/+^ mice 6 months after injection (Fig. 2A), but, in the absence of Gal-3, fibrosis was significantly disorganized showing dispersed collagen deposition throughout the submesothelium and adipose tissue (Fig. 2B). Gal-3^+^ cells were identified in the mesentery of Lgals3^+/+^ mice treated with pristane, associating the presence of Gal-3 with fibrosis. These cells were preferentially located in the submesothelial regions, specially surrounding fat-storage plates (Fig. 2C). As expected, cells expressing Gal-3 were not identified in Lgals3^−/−^ mice (Fig. 2D). Although histological alterations have been substantially observed in the absence of Gal-3, the percentage of annexin-V^+^PI^+^ and annexin-V^+^PI^−^ apoptotic cells was similar between mesenteric cells of Lgals3^+/+^ and Lgals3^−/−^ mice. However, the percentage of annexin-V^−^PI^+^ dead mesenteric cells was significantly increased in Lgals3^−/−^ mice treated with pristane (Supplementary Fig. 1). These data suggested that Gal-3 can be a critical molecule to organize fibrotic reactions but apparently did not interferes with cell death dependent on Annexin-V staining.

### Absence of Gal-3 partially interfered macrophage polarization in the mesentery of pristane-treated mice

**Figure 3:**
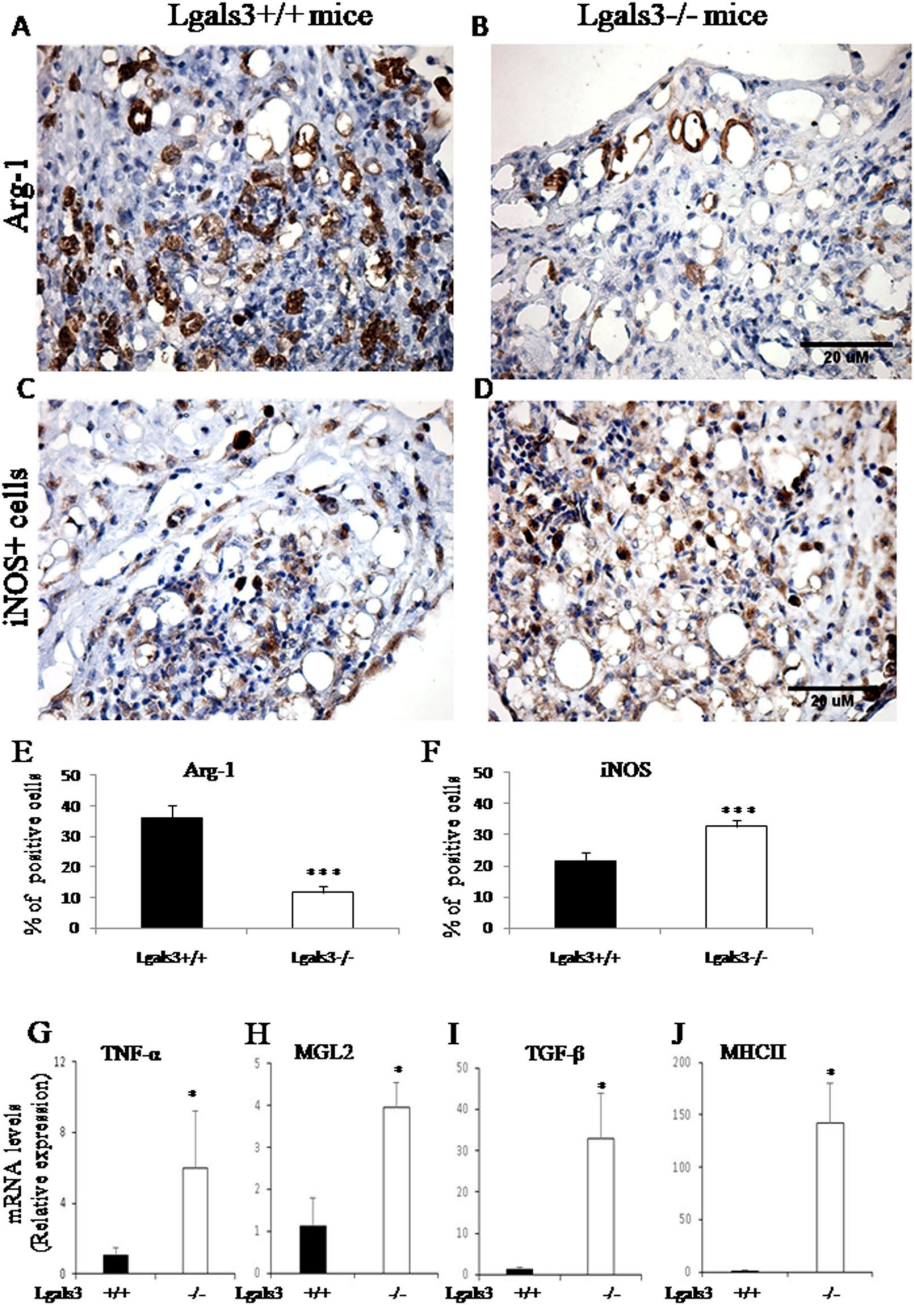
Analysis of M1/M2 markers on pristane-induced mice. Arginase-1 (M2 marker) was evaluated in mesenteric cells of pristane-induced Lgals3^+/+^ mice (A) and Lgals3^−/−^ mice (**B**). Arrows pointed to crown-like structures positive to Arg-1 in both mice groups. (**C** and **D**) iNOS (M1 marker) was detected in pristane-induced Lgals3^+/+^ and Lgals3^+/+^ mice, respectively. Bar graphs indicate the percentage of Arg-1^+^ cells (**E**) and iNOS^+^ cells (**F**). By real-time PCR, the expression of M1 genes (TNF-α and MGL-2) and M2 genes (TGFβ1 and MHCII) was monitored and relative expression was plotted in graphs (G-J, respectively). Black bars represent Lgals3^+/+^ mice whereas white bars represent Lgals3^−/−^ mice. Data are representative of three independent experiments. Magnification: A-D (200×). (*) indicates p < 0.05. (***) indicates p < 0.001.

The hallmarked presence of Gal-3^+^ cells surrounding lipid-like structures was directly related to F4/80^+^ macrophages (Supplementary Fig. 2), suggesting a critical role for Gal-3 during macrophage responses to pristane. As consequence, we analyzed the macrophage polarization and observed a significant imbalance between M1/M2 markers. Clearly, Arginase-1^+^ cells were preferentially located surrounding fat-storage niches, similar with Gal-3 stain, indicating a direct correlation with crown-like structures, in both Lgals3^+/+^ and Lgals3^−/−^ mice (Fig. 3A mB, arrows). In contrast, iNOS^+^ cells were scarcely associated with these crown-like structures in these mice (Fig. 3C,D). The proportion of these cells was changed in the absence of Gal-3. In the mesentery of Lgals3^−/−^ mice induced by pristane, the percentage of Arg-1^+^ cells were significantly reduced in comparison with respective Lgals3^+/+^ mice (Fig. 3E). In contrast, the percentual of iNOS^+^ cells was higher Lgals3^−/−^ than Lgals3^+/+^ mice both induced by pristane (Fig. 3F). Mesenteric tissues of Lgals3^+/+^ and Lgals3^−/−^ mice not treated with pristane were negative to Arg-1 and iNOS staining (Supplementary Fig. 3A–D, respectively). Suggestively, galectin-3 seems to organize iNOS^+^ and Arg-1^+^ cell niches during inflammatory response in the mesentery.

The immunohistochemistry revealed a potential modulation of macrophage polarization dependent of Gal-3 in the mesentery affected by pristane reaction. Indeed, the real time-PCR analysis indicated that Gal-3 controls TNF-α, TGF-β1, MHCII and MGL2 gene expression by mesenteric cells. It was found that the expression of these genes was significantly increased in the mesentery of Lgals3^−/−^ mice when compared with Lgals3^+/+^ mice, both groups induced by pristane (Fig. 3G–J). Data showed that M1 markers (TNF-α and MGL-2) and M2 markers (TGF-β and MHCII) were overexpressed in the absence of Gal-3. Together, these results suggested that Gal-3 organizes macrophage niches in the mesentery and controls the expression of genes associated with M1 and M2 polarization during inflammatory and fibrogranulomatous reaction induced by pristane.

### Monocyte recruitment is impaired in early events associated to pristane injection in Lgals3^−/−^ mice

In order to investigate whether leukocyte recruitment and macrophage polarization, we analyzed pristane-induced mice 18 h after the i.p. injection to evaluate early inflammatory events. Hematological parameters and peritoneal floating cells were collected and quantified in all mice groups. The number of red blood cells (RBCs) did not change after treatment with pristane in Lgals3^+/+^ (controls: 6.9 × 10^6^ ± 2.6 RBCs/mm^3^; Pristane: 8.3 × 10^6^ ± 1.4 RBCs/mm^3^). However, RBCs of Lgals3^−/−^ mice were numerically different 18 h after the injection of pristane (controls: 10.6 × 10^6^ ± 1.9 RBCs/mm^3^; Pristane: 16.3 × 10^6^ ± 2.6 RBCs/mm^3^) (Table 1). On the other hand, the number of white blood cells (WBCs or leukocytes) increased in both Lgals3^+/+^ (ctr: 9.2 × 10^3^ ± 1.7 WBCs/mm^3^; Prist: 22.4 × 10^3^ ± 3.1 WBCs/mm^3^) and Lgals3^−/−^ (ctr: 5.3 × 10^3^ ± 0.9 WBCs/mm^3^; Prist: 14.9 × 10^3^ ± 3.6 WBCs/mm^3^) mice groups after treatment with pristane (Table 1). Platelet numbers (Plats) were significantly reduced after pristane-induction in both Lgals3^+/+^ (ctr: 198.4 × 10^3^ ± 32.2 Plats/mm^3^; Prist: 112.6 × 10^3^ ± 28.2 Plats/mm^3^) and Lgals3^−/−^ (ctr: 98.8 × 10^3^ ± 12.3 Plats/mm^3^; Prist: 37.4 × 10^3^ ± 10.6 Plats/mm^3^) mice (Table 1).

**Table 1:** Hematological parameters in the blood 18 h after pristane injection.

Blood (cells/mm^3^)	Ctr Lgals3^+/+^	Prist Lgals3^+/+^	Ctr Lgals3^−/−^	Prist Lgals3^−/−^
Red blood cells	6.9 × 10^6^ (±2.6)	8.3 × 10^6^ (±1.4)	10.6 × 10^6^ (±1.9)	16.3 × 10^6^ (±2.6)^*#^
White blood cells	9.2 × 10^3^ (±1.7)	22.4 × 10^3^ (±3.1)*	5.3 × 10^3^ (±0.9)	14.9 × 10^3^ (±3.6)^*#^
Platelets	198.4 × 10^3^ (±32.2)	112.6 × 10^3^ (±28.1)*	98.8 × 10^3^ (±12.3)	37.4 × 10^3^ (±10.6)^*#^

Ctr: control mice; Prist: pristane-injected mice.

(*) indicates p < 0.05 to pristane-treated mice compared with respective controls.

(^#^) indicates p < 0.05 to pristane-induced Lgals-3^−/−^ mice compared with pristane-induced Lgals-3^+/+^ mice.

The peritoneal cellularity was significantly increased in Lgals-3^+/+^ and Lgals-3^−/−^ mice treated pristane, although the number of peritoneal cells was lower in the absence of Gal-3 (Table 2). Interestingly, the proportion of cells recruited to peritoneal cavity was similar between both mice groups. The ratio of pristane-Lgals3^+/+^ by control-Lgals3^+/+^ (26.1/8.2 cells per mL) indicated that peritoneal cellularity increased 3.18-fold 18 h after pristane-injection (Table 1). On the other hand, pristane-Lgals3^−/−^ by control-Lgals3^−/−^ ratio (11.4/3.7 cells per mL) indicated that peritoneal cellularity increased 3.08-fold 18 h after pristane-injection (Table 2). These data suggested that recruitment index to peritoneal cavity was similar between Lgals3^+/+^ and Lgals3^−/−^ both induced by pristane.

**Table 2:** Number of cells in the peritoneal fluid 18 h after pristane injection.

Peritoneal fluid	Ctr Lgals3^+/+^	Prist Lgals3^+/+^	Ctr Lgals3^−/−^	Prist Lgals3^−/−^
Cellularity (cells/mL)	8.2 × 10^6^ (±0.8)	26.1 × 10^6^ (±5.9)*	3.7 × 10^6^ (±0.6)	11.4 × 10^6^ (±1.2)*^#^
Granulocytes (cells/mL)	1.6 × 10^5^ (±0.5)	9.8 × 10^5^ (±1.1)*	0.5 × 10^5^ (±0.8)	11.3 × 10^5^ (±2.5)*
T lymphocytes (cells/mL)	1.9 × 10^6^ (±0.7)	2.1 × 10^6^ (±0.9)	2.6 × 10^6^ (±0.7)	2.9 × 10^6^ (±1.1)
B lymphocytes (cells/mL)	13.4 × 10^6^ (±2.9)	14.8 × 10^6^ (±2.1)	5.9 × 10^6^ (±1.4)	6.3 × 10^6^ (±1.9)^#^

Ctr: control mice; Prist: pristane-injected mice.

(*) indicates p < 0.05 to pristane-treated mice compared with respective controls.

(^#^) indicates p < 0.05 to pristane-induced Lgals-3^−/−^ mice compared with pristane-induced Lgals-3^+/+^ mice.

**Figure 4:**
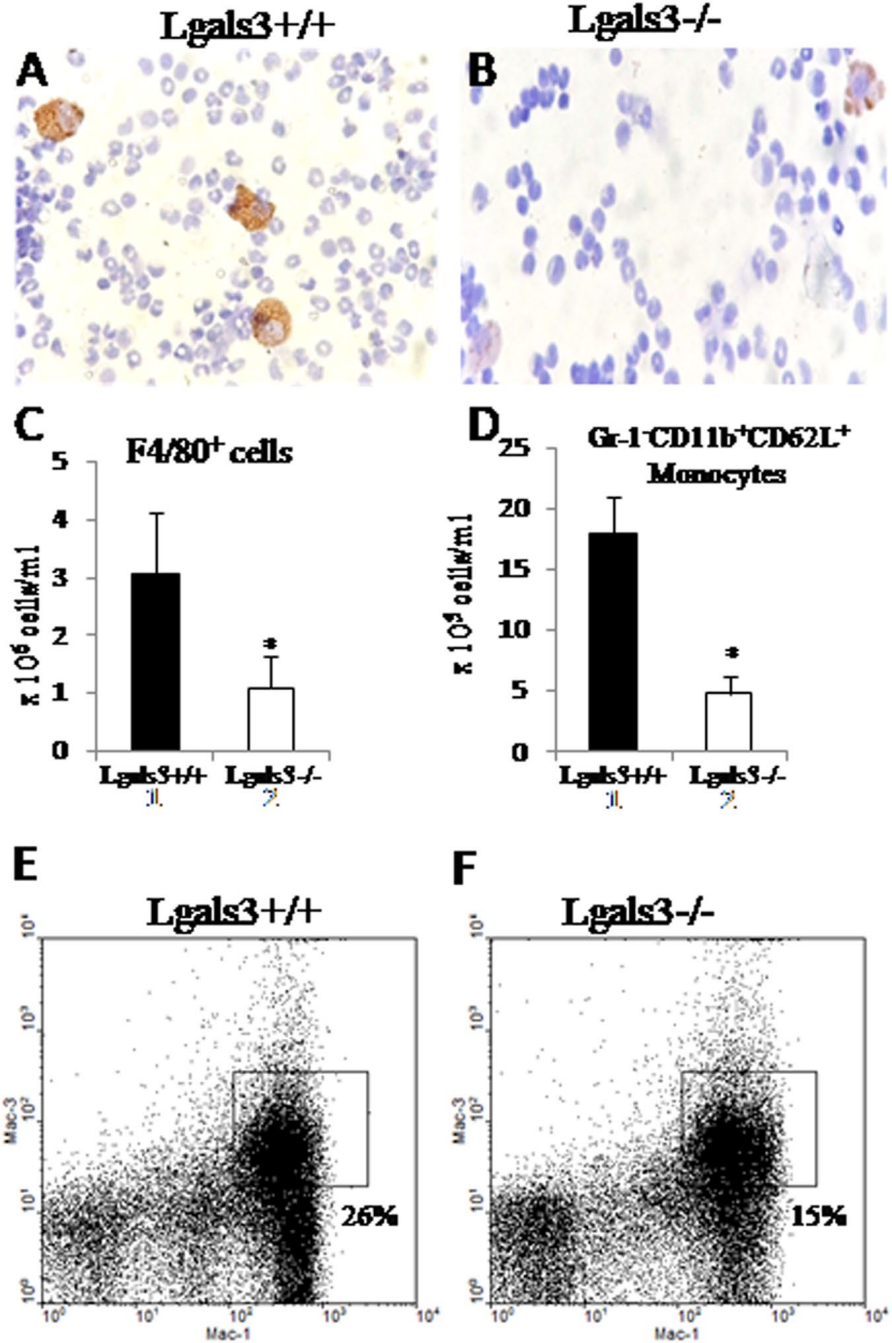
Analysis of peritoneal monocytes and macrophages recovered 18 h after pristane injection. Immunocytochemistry to identify F4/80^+^ peritoneal cells in cytosmears of pristane-induced Lgals3^+/+^ mice (**A**) and Lgals3^−/−^ mice (**B**). Bar graphs indicate the absolute number of F4/80^+^ cells (**C**) and Gr-1^-^CD11b^+^CD62L^+^ monocytes (**D**) in the peritoneal fluid. Black bars represent Lgals3^+/+^ mice whereas white bars represent Lgals3^−/−^ mice. Total phagocytes were quantified by flow cytometry according co-expression of Mac-1/Mac-3 in Lgals3^+/+^ mice (**E**) and Lgals3^−/−^ mice (**F**). Data are representative of three independent experiments. Magnification: A-B (200×). (*) indicates p < 0.05.

Peritoneal macrophages expressing F4/80 were strongly stained in pristane-induced Lgals3^+/+^ (Fig. 4A) and poorly detected in the peritoneal fluid of pristane-induced Lgals3^−/−^ mice (Fig. 4B), both 18 h after injection. The number of these cells expressing F4/80 was significantly reduced in Lgals3^−/−^ (Fig. 4C). Recruited monocytes (Gr-1^−^CD11b^+^CD62L^+^) were significantly reduced in the peritoneal cavity of pristane-induced Lgals3^−/−^ mice when compared with Lgals3^+/+^ mice (Fig. 4D). Moreover, the percentage reduction of peritoneal Mac-1^+^Mac-3^+^ phagocytic cells on 18 h-pristane-induced Lgals3^−/−^ mice in comparison with Lgals3^+/+^ mice (Fig. 4E,F), reinforced that monocyte recruitment impaired in the absence of Gal-3 modified early events associated to pristane induction, such as phagocytic capacity.

As cellularity was increased after pristane injection, but monocyte/macrophage lineages were reduced, we analyzed other cell types. The number of recruited granulocytes (Gr-1^+/high^CD11b^+^CD62L^+^ cells) increased in both mice groups 18 h after pristane injection (Table 2). T lymphocytes (B220^−^CD5^+^ cells) were numerically similar at this time, independent on mice group (Table 2). On the other hand, B lymphocytes (B220^+^CD5^+/−^ cells) were increased in Lgals3^+/+^ when compared with Lgals3^−/−^ mice in both condition, control and induced with pristane (Table 2).

### Absence of Gal-3 disorganized peritoneal B cell compartments and mesenteric niches in mice influenced by pristane

**Figure 5:**
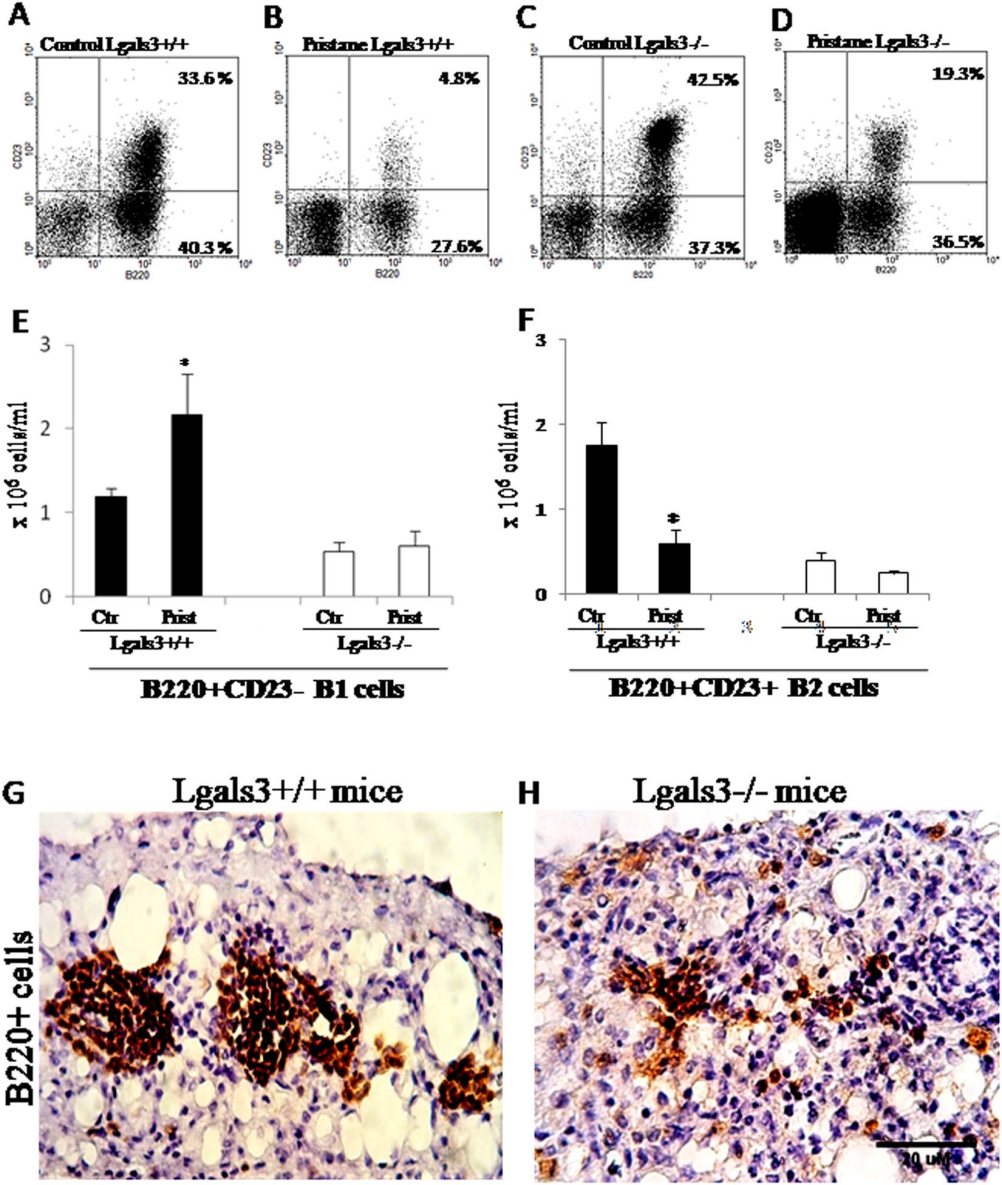
Phenotype of peritoneal-floating B cells and their niches in the mesentery of pristane-induced mice. Dot plot graphs showing B cell subpopulations in the peritoneal fluid of Lgals3^+/+^ (**A** and **B**, control and pristane) and Lgals3^−/−^ mice (**C** and **D**, control and pristane). Bar graphs indicate the absolute number of peritoneal B220^+^CD23^-^ B1 cells (**E**) and B220^+^CD23^+^ B2 cells (**F**) in all experimental mice induced by pristane for 18 h. On month 6, mesenteric B220^+^ cells niches were restricted to organized clusters on Lgals3^+/+^ (**G**) and dispersed in Lgals3^−/−^ mice (**H**). Black bars represent Lgals3^+/+^ mice whereas white bars represent Lgals3^−/−^ mice 18 h after pristane treatment and respective controls. Data are representative of three independent experiments. G-H: magnification 200×. (*) indicates p < 0.05.

The imbalance of monocyte/macrophage populations in response to pristane 18 hours and 6 months after injection led us to investigate B cell subpopulations in the peritoneal cavity in Lgals3^−/−^ mice. B1 (B220^+^CD23^−^) and B2 (B220^+^CD23^+^) lymphocytes were monitored by flow cytometry. It is important to note that B cell subpopulations respond to pristane treatment^27^. The percentage of B220^+^CD23^−^ B1 and B220^+^CD23^+^ B2 lymphocytes in both pristane-induced Lgals3^+/+^ and Lgals3^−/−^ mice changed after pristane treatment (Fig. 5A–D). Analysis of absolute number of B lymphocytes revealed that B220^+^CD23^−^ B1 cells were significantly increased in Lgals3^+/+^ pristane-induced mice, but no changes were detected in B1 cell compartment of Lgals3^−/−^ mice (Fig. 5E). On the other hand, B220^+^CD23^+^ B2 lymphocytes were significantly reduced in Lgals3^+/+^ pristane-induced mice. Newly, no changes were observed in Lgals3^−/−^ mice (Fig. 5F).

The imbalance on B cell response to early inflammatory events in contact with pristane led us to study B cell niches in the mesentery after 6 months of pristane exposition. The presence of B220^+^ cells reinforced our previous data about organization of milky spots in the mesentery stimulated by pristane. In Lgals3^+/+^ mice, B220^+^ cells were markedly present in well-defined milky spots (Fig. 5G). In the absence of Gal-3, these B lymphocytes were widely dispersed throughout the mesentery (Fig. 5H), indicating a substantial disorganization of milky spots in the mesentery of Lgals3^−/−^ pristane-induced mice. Consistently, mesenteric tissues of Lgals3^+/+^ and Lgals3^−/−^ mice not treated with pristane were negative to B220 immunostaining (Supplementary Fig. 3E,F).

**Figure 6:**
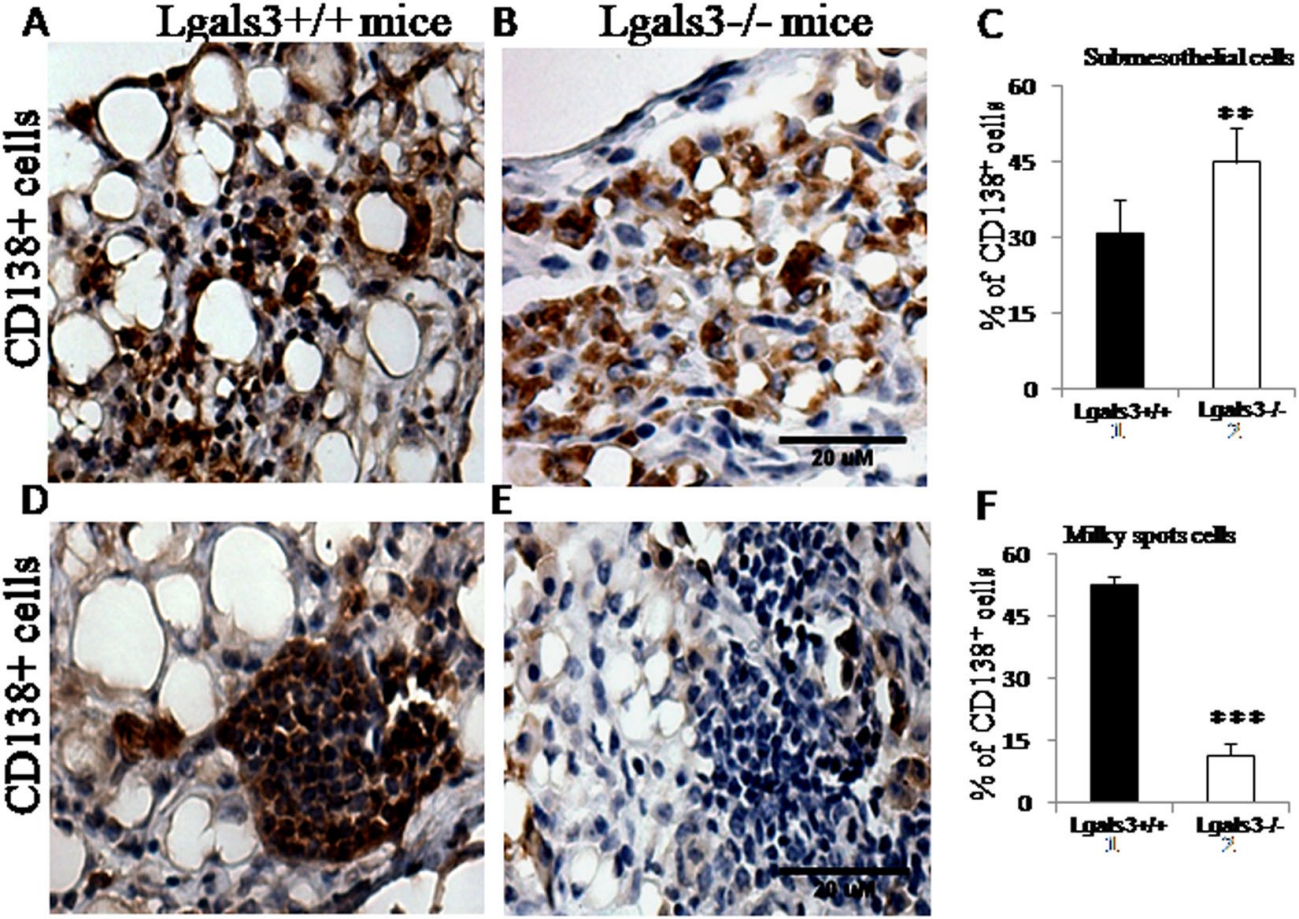
Immunohistochemistry to detect B lymphocyte and plasma cell niches in mesentery of pristane-induced mice. CD138^+^ cells localized in non-lymphoid submesothelial regions of Lgals3^+-+^ (**A**) and Lgals3^−/−^ mice (**B**). These cells were quantified and plotted in graph bars (**C**). CD138 + cells observed in milky spots of Lgals3^+/+^ (**D**) and Lgals3^−/−^ mice (**E**). Bar graphs indicate the percentage of CD138^+^ cells in milky spots (**F**). Black bars represent Lgals3^+/+^ mice whereas white bars represent Lgals3^−/−^ mice. Magnification: A-D (500×). (**) indicates p < 0.01. (***) indicates p < 0.001.

Morphological changes in the milky spots and B cell niches indicated that plasma cell organization could be affected in the absence of Gal-3 6 months after pristane treatment. The distribution of submesothelial CD138^+^ plasma cells was severely changed pristane-induced Lgals3^−/−^ mice when compared with Lgals3^+/+^ mice (Fig. 6A,B). The percentage of these cells were significantly increased in the absence of Gal-3 (Fig. 6C). In the milky spots, CD138^+^ cells were well-clustered in the mesentery of pristane-induced Lgals3^+/+^ mice, but poorly observed in Lgals3^−/−^ mice (Fig. 6D,E). The percentage of these cells was significantly reduced in the absence of Gal-3 (Fig. 6F). The mesentery of Lgals3^+/+^ and Lgals3^−/−^ mice not treated with pristane were negative to CD138 immunostaining (Supplementary Fig. 3G,H).

### Notch/Delta signaling is significantly modified in pristane-induced Lgals3^−/−^ mice

**Figure 7:**
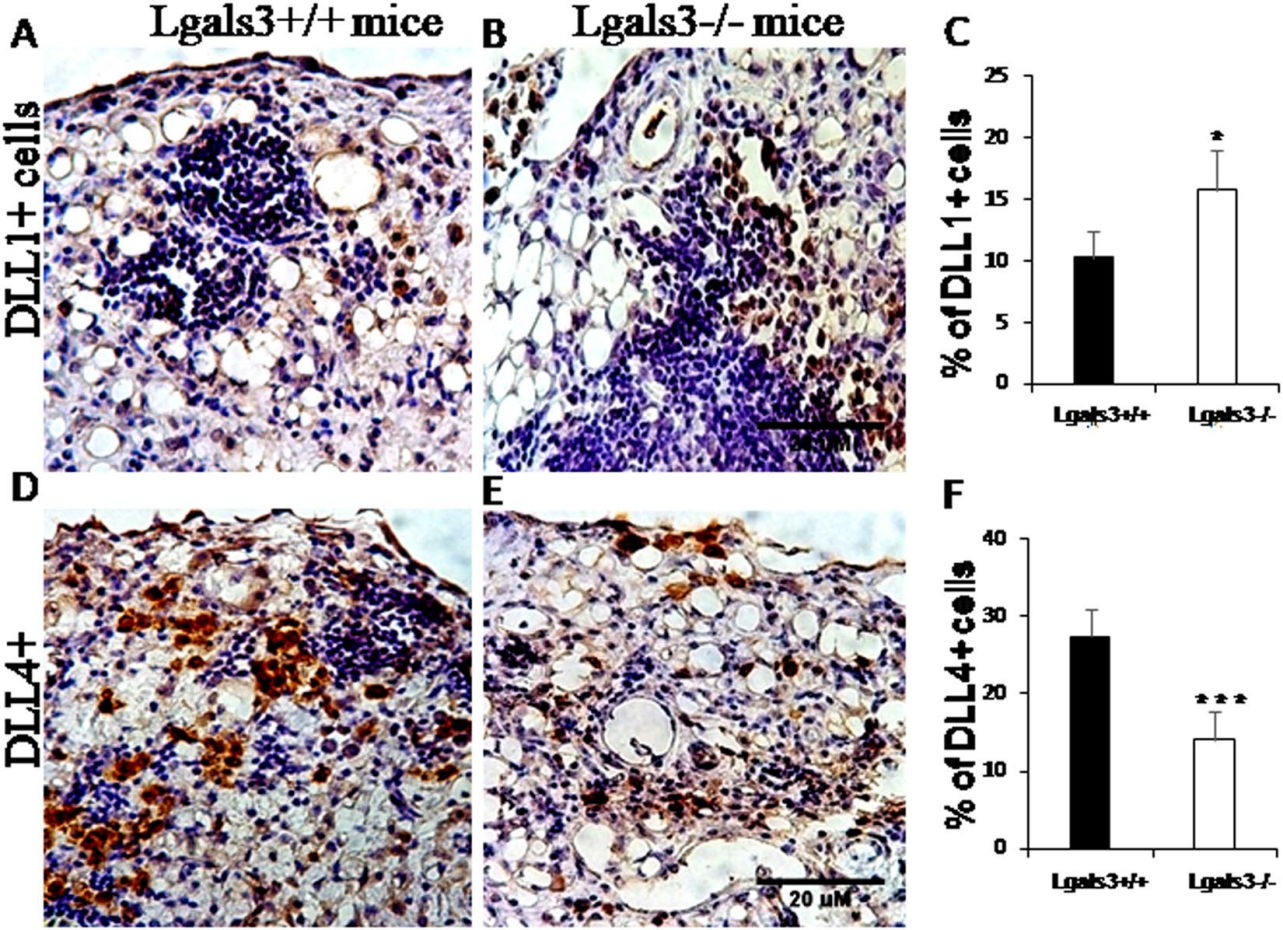
Immunohistochemistry to Notch ligands on pristane-induced mice. Delta-like-1 (DLL1) was markedly present in cells surrounding milky spots in Lgals3^+/+^ induced by pristane (**A**). In Lgals3^−/−^, these cells were randomly dispersed through mesentery (**B**). Bars graphs indicate the percentage of DLL1^+^ cells (**C**). Delta-like-4 (DLL4) expressing cells were found in clusters dispersed by submesothelium region in Lgals3^+/+^ (**D**). In Lgals3^−/−^, these cells were observed in all compartments of the mesentery (**E**). The quantity of cells was plotted in graph bars (**F**). Black bars represent Lgals3^+/+^ mice whereas white bars represent Lgals3^−/−^ mice. Data are representative of three independent experiments. Magnification: A-D (200×). (*) indicates p < 0.05. (***) indicates p < 0.001.

Recently, we demonstrated that Gal-3 interferes with Notch/Delta signaling in lymphoid tissues and B cell differentiation into plasma cells^28^. Considering that B lymphocytes and plasma cell niches were drastically disturbed in the mesentery 6 months after pristane stimuli, it was plausible to investigate the role of Notch/Delta pathways in our model. In both mice groups, DLL1^+^ cells were preferentially located surrounding milky spots (Fig. 7A,B). However, the percentage of these DLL1^+^ cells was significantly increased in Lgals3^−/−^ mice (Fig. 7C). Samples obtained from Lgals3^+/+^ and Lgals3^−/−^ mice not treated with pristane were negative to DLL1 staining (Supplementary Fig. 3I,J).On the other hand, DLL4^+^ cells were randomly distributed throughout the mesenteric tissues (Fig. 7D,E) and percentage of these cells was significantly decreased in Lgals3^−/−^ mice (Fig. 7F). Furthermore, mesenteric tissues of Lgals3^+/+^ and Lgals3^−/−^ mice not treated with pristane were negative to DLL4 immunostaining (Supplementary Fig. 3K,L).

In order to study a possible early mechanism to explain the disturbances on B cell niches, we returned to 18 hours post-injection to observe the pattern of distribution of dendritic cells (DC). Pristane induced a significant increase of B220^+^Gr-1^+^CD11c^+^CD11b^−^ plasmacytoid DC (pDC) and B220^−^Gr-1^−^CD11c^+^CD11b^+^ classic DC (cDC) in the peritoneal fluid of both mice groups. However, pDC was numerically increased in Lgals3^−/−^ while cDC were significantly reduced in Lgals3^−/−^ mice, when compared with Lgals3^+/+^ mice (Table 3). Together, these data indicated that mesenteric B lymphocyte and plasma cell niches are widely disorganized in Lgals3^−/−^ mice due to pDC polarization in early events associated to pristane induction that result in significant imbalance in DLL1/DLL4-Notch signaling and macrophage M1/M2 differentiation. We suggested these mechanisms as high potential to be investigated in experimental lupus disease.

**Table 3:** Number of DC in the peritoneal fluid 18 h after pristane injection.

Peritoneal fluid	Ctr Lgals3^+/+^	Prist Lgals3^+/+^	Ctr Lgals3^−/−^	Prist Lgals3^−/−^
pDC (cells/mL)	1.4 × 10^3^ (±0.6)	22.4 × 10^3^ (±4.7)*	1.7 × 10^3^ (±0.9)	58.4 × 10^3^ (±8.9)*^#^
cDC (cells/mL)	3.6 × 10^3^ (±1.1)	37.6 × 10^3^ (±7.3)*	0.9 × 10^3^ (±0.4)	19.9 × 10^3^ (±6.4)*^#^

Ctr: control mice; Prist: pristane-injected mice.

(*) indicates p < 0.05 to pristane-treated mice compared with respective controls.

(^#^) indicates p < 0.05 to pristane-induced Lgals-3^−/−^ mice compared with pristane-induced Lgals-3^+/+^ mice.

**Figure 8:**
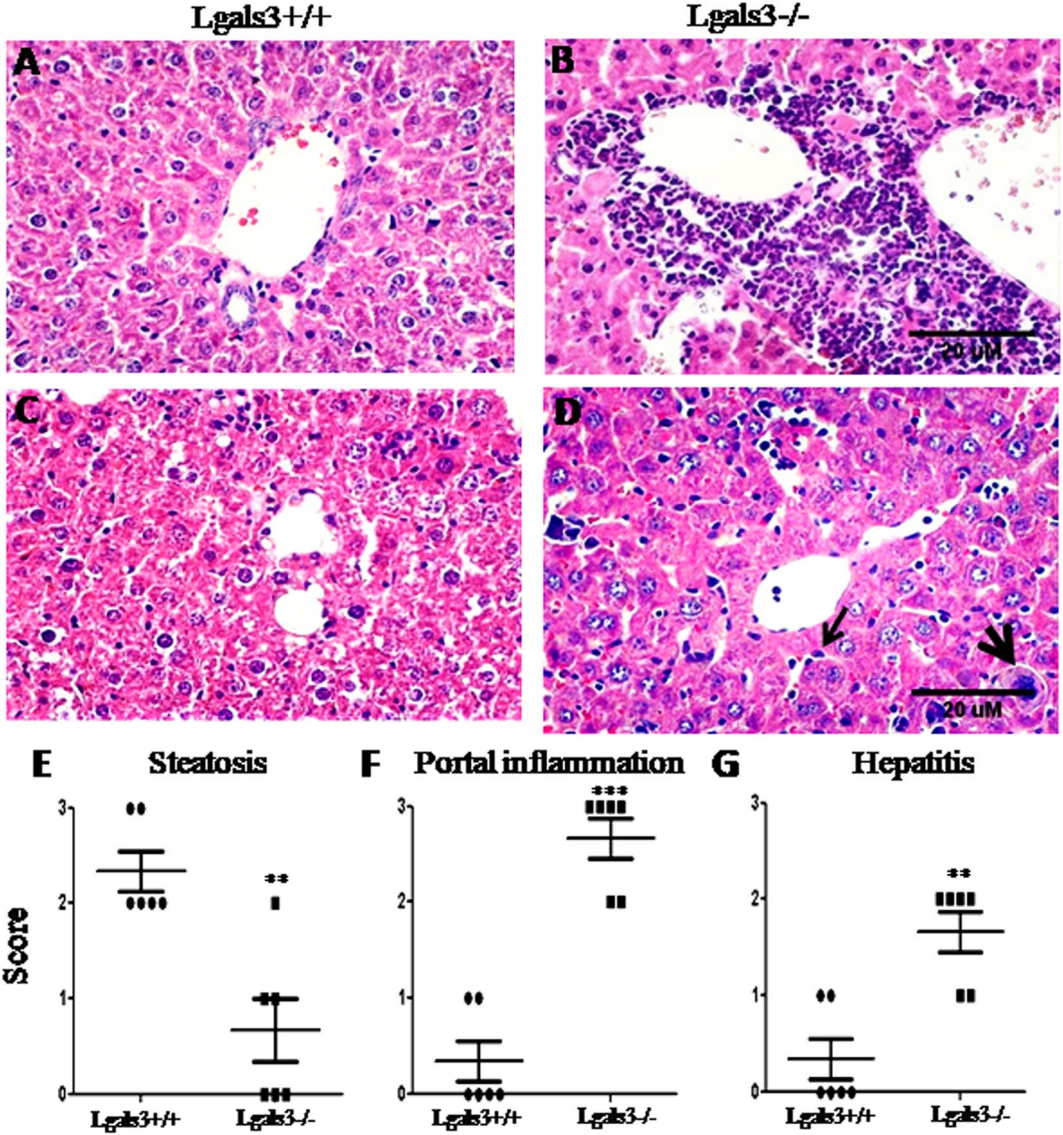
Photomicrographs of liver on pristane-induced mice. Representative images of livers obtained from Lgals3^+/+^ and Lgals3^−/−^ mice both treated with pristane for 6 months. (**A**) Portal zone of Lgals3^+/+^ mice showing absence of inflammation. (**B**) Intense inflammatory infiltrate in the portal zone of Lgals3^−/−^ mice. (**C**) Lobular zone of Lgals3^+/+^ mice showing steatosis *foci*. (**D**) Lobular zone of Lgals3^−/−^ mice showing plasma cells (arrow) and megakaryocytes (arrowhead). Score to steatosis (E), portal inflammation (**F**) and hepatites (**G**) were plotted in bar graphs. Black bars indicate Lgals3^+/+^ mice and white bars represent Lgals3^−/−^ mice. Data are representative of three independent experiments. Magnification: A-D (100×) and E-F (500×).

These findings in the mesentery organization, widely disturbed in the absence of Gal-3, led us to investigate the liver considering the natural influx of mesenteric compounds to hepatic portal zones. The liver of Lgals3^−/−^ mice presented severe portal inflammation characterized by granulocyte and mononuclear infiltrate 6 months after the pristane treatment (Fig. 8A,B). In the lobular zone, hepatic steatosis was predominant in the Lgals3^+/+^ mice whereas atypical cells (such as plasma cells and megakaryocytes) were frequently observed in dilated sinusoids in the liver of Lgals3^−/−^ mice (Fig. 8C,D). The scores of hepatic steatosis (higher in Lgals3^+/+^ mice), portal inflammation and hepatitis (both higher in Lgals3^−/−^ mice) indicated that liver injuries were aggravated in the absence of Gal-3 (Fig. 8E–G).

## Discussion

For the first time, we demonstrated that Gal-3 regulates inflammatory reaction in the mesentery during lupus-like responses induced by pristane. As reasonable, it was demonstrated that pristane treated Lgals3^+/+^ mice have significant submesothelial fibrosis correlated with high concentration of Gal-3^+^ cells, organized milky spots, and predominant M2 macrophage polarization. In contrast, pristane-induced Lgals3^−/−^ mice showed severe diffuse fibrosis throughout submesothelium and adipose tissue, disorganized clusters of B lymphocytes and plasmas cells, and M1 macrophage polarization associated with imbalanced DLL-1 and DLL-4 response. All these data can be associated with liver damages characterized by severe hepatitis in mice knockout for Gal-3 in lupus-like syndrome induced by pristane.

Mesentery was recently described as organ^25^ and its involvement with pathogenesis of autoimmune diseases is poorly understood. In this experimental lupus-like model, pristane is inoculated inside of the peritoneal cavity promoting early responses in mesenteric and peritoneal cells that culminate in granuloma formation, intense lymphoid and myeloid cells recruitment/activation and autoantibodies secretion^26,27,29^. Recently, we demonstrated important changes on granuloma formation in the absence of Gal-3^7^, using a pristane-based protocol that causes mouse plasmacytoma, applying pristane i.p. each 60 days in the course of 180 days^30^. In this work, we explore non-granulomatous regions in the mesentery submitted to a single injection of pristane in the peritoneal cavity (lupus-like experimental condition)^22^.

Pristane induces lupus-like disease six months after a single intraperitoneal injection^22^, including autoantibody repertoire and other clinical markers^26^. Classical aspects examined to lupus diagnosis were neglected in our work, such as proteinuria and anti-c1q. Our major point was to investigate the mesentery and liver in the context of lupus-like syndrome defined by pristane. Lgals3^**+/+**^ mice presented typical mesenteric tissues: mesothelium, submesothelium and adipose tissue. Although submesothelium showed significant inflammatory infiltrate and moderate fibrosis, these histological compartments were well-preserved when compared with non-treated mice. In contrast, Lgals3^−/−^ mice showed drastic mesenteric disorganization associated with diffused submesothelial fibrotic reaction, atypical plasma cells and intense influx of polymorphonuclear cells. It is not clear whether morphological findings indicate a possible continuity of inflammatory triggers in the absence of Gal-3 leading to architectural differences or poor resolution of inflammatory status defined by the presence of pristane.

The submesothelial reactive response to tissue damages has been described in distinct models^31–33^. Recently, our group demonstrated that submesothelial cells derived from peritoneal explants have fibroblast-like behavior, mesenchymal phenotype, and stromal functions^34,35^. The pristane-model was marked by important disturbances on oil granuloma formation in Lgals3^−/−^ mice, including large necrotic areas and high levels of IL-6^7^. However, the role of submesothelial Gal-3^+^ to drive autoimmune response continue unclear.

Fibrotic sites can be organized by Gal-3 in different experimental conditions. In the liver, Henderson and colleagues showed that Gal-3 is critical to myofibroblast activation and hepatic fibrosis^5^. Our group revealed that Lgals3^−/−^ mice have dispersive fibrosis in the liver linked to severe intra-hepatic myelopoiesis during chronic *Schistosoma mansoni* infection^36^. In the kidney, Gal-3 secreted by macrophages promotes renal fibrosis^37^. In this context, Chen and colleagues reviewed important concepts joining high levels of plasma Gal-3 and progressive renal impairment^6^. Here, we detected strong Gal-3 staining colocalizing with fibrosis in mesenteric submesothelium regions. However, collagen fibers were significantly dispersed throughout submesothelium and adipose tissue of Lgals3^−/−^ mice. Then, we suggested that Gal-3 plays a regulatory role on fibrotic sites in the mesentery.

In autoimmune diseases, including SLE, Gal-3 has been attracted the attention for multiple functions^13^. The high level of Gal-3-binding protein in circulating cell-derived microparticles has been markedly observed in SLE patients. Although no association with clinical manifestations was detected, it was observed an intriguing formation of immune complexes with IgG in lupus nephritis biopsies^38^. In lupus glomerulonephritis, Gal-3 has been investigated as prognostic biomarker and promising therapeutic target^16^. Pristane-induced lupus model has been used to study the etiology related to non-functional B and T cell responses, autoantibody production, and multiple tissue damages. Currently, this experimental model is considered excellent to understand mechanisms that result in autoimmunity^22^. In this work, we found that T cell compartments were poorly changed in Lgals3^−/−^ mice induced with pristane. In contrast, B cell niches were severely modified in the absence of Gal-3, at least in part, interfering with B lymphocyte differentiation into plasma cells. In these pristane-induced Lgals3^−/−^ mice, plasma cells were significant disorganized in the mesentery and possibly involved with the pathogenesis of experimental SLE.

The question about mechanisms that pristane induces lupus remains unclear. However, evidence-based on loss of immune tolerance started by incoherent programmed cell death or by an exposition to a storm of cytokines in inflammatory microenvironments. One of cellular target is macrophage, a critical cell type for development of inflammation and tissue repair. Pristane i.p. injected in mice recruits inflammatory monocytes to peritoneal cavity by INF-type I and CCR2 dependent manners^39^. In this work, we found that Gal-3 favors influx of inflammatory monocytes 6 months after pristane injection, indicating that these cells are persistently recruited to peritoneal cavity in this model. Interestingly, in Lgals3^−/−^ mice, pristane weakly induced the migration of inflammatory monocytes to peritoneal cavity.

Macrophages activated classically (M1 macrophages) synthetize pro-inflammatory proteins, including IFN-γ, IL-1β, MGL-2 and TNF-α. On the other hand, alternatively activated macrophages (M2 macrophages) produce anti-inflammatory proteins such as IL-4, IL-10, MHCII and TGF-β^40^. Another phenotype distinction is based on the balance of iNOS (M1) and ARG-1 (M2) expression^41^. Gal-3 was critical to M1 to M2 transition^42^. Our data indicated that Lgals3^−/−^ mice showed increased number of iNOS^+^ cells and reduced number of Arg-1^+^ cells compared to Lgals3^+/+^ mice 6 months after the injection with pristane. It was plausible to propose that tendency M1 polarization in Lgals3^−/−^ mice delayed the tissue repair and amplified the inflammatory reaction. Considering that Lgals3^+/+^ and Lgals3^−/−^ control mice (non-induced with pristane) did not show iNOS^+^ and Arg-1^+^ cells in mesentery, it is plausible to suggest that mesenteric inflammatory reaction was driven by M1 and M2 stimuli and the absence of Gal-3 amplified both mechanistic signals orchestrating the macrophage polarization. By Immunohistochemistry and real-time PCR, we observed that M1 (iNOS, TNF-α and MGL-2) and M2 (ARG-1, MHCII and TGF-β1) markers were substantially increased in both Lgals3^+/+^ and Lgals3^−/−^ pristane-induced mice, indicating promiscuous effects of Gal-3 during M1/M2 polarization in the mesentery. These data pointed to Gal-3 as potent tissue organizer and its absence generated an atypical macrophage polarization in lupus-like syndrome.

Notch pathways also interfere with M1/M2 polarization^43^. DLL4/Notch impairs Arg-1^+^ M2 differentiation by mechanisms that include M1 macrophage differentiation and inhibition of M2 gene expression^44^. In addition, Notch signaling is disturbed in the absence of Gal-3^45^. Recently, we demonstrated that Lgals3^−/−^ mice presented elevated number of splenic and bone marrow DLL4^+^ cells in comparison to Lgals3^+/+^ mice^28^. However, in this work we found that pristane-induced Lgals3^−/−^ mice showed lower frequency of DLL4^+^ cells compared to respective Lgals3^+/+^ mice. Importantly, study in lymphoid tissues was performed in C57BL/6 mice while here we used BALB/c mice.

Notch/Dll1 also regulates humoral immune responses inducing B lymphocyte differentiation into plasma cells^46^. Here, we observed that Lgals3^−/−^ mice induced with pristane showed higher numbers of DLL1^+^ cells in the mesentery compared to respective control Lgals3^+/+^ mice with BALB/c background. Again, these data were opposites of those obtained with C57BL/6 mice^28^. Consistently, Gal-3 was described in anergic B cells of BALB/c mice^17^, but absent in non-activated B lymphocytes in C57BL/6 mice^12^. Together, these finds pointed to distinct functions of Gal-3 on B cell differentiation depending on genetic background of mice.

The recent paper of Beccaria and colleagues reinforced the use of BALB/c mice to study of Gal-3 in experimental lupus. They revealed that Lgals3^−/−^ mice naturally developed lupus-like disease by controlling germinal center responses via IFN-γ^17^. In accordance, in the absence of Gal-3, we observed some hematological parameters compatible with SLE, including significant thrombocytopenia^47^. Here, we used a classic model to promote lupus-like disease in mice by pristane^22^. To understand early cellular events in this experimental model, we analyzed some cell types within peritoneal cavity 18 h after the injection. Besides on thrombocytopenia, Lgals3^−/−^ mice showed erythrocytosis and leukopenia when compared with Lgals3^+/+^ mice both treated with pristane. The imbalance between classic (myeloid) DC and plasmacytoid DC can be responsible for disturbances described in the mesentery. Although Ly6G^high^ immature monocytes have considered the major source of IFN-I in this experimental lupus-model, DC play important roles in the pathogenesis of lupus in tertiary lymphoid tissues^48,49^. Here, we found that DC of Lgals3^+/+^ were a mixed population containing similar numbers of mDC and pDC. On the other hand, the number of pDC was significantly increased in Lgals3^−/−^ 18 h after pristane induction. These data indicate a putative cellular mechanism driving the aberrant B cell response in the absence of Gal-3.

One significant consequence of mesenteric disturbances detected in our experimental model was lupoid hepatitis. The involvement of liver typically occurs 10–11 months after the single injection of pristane and the major histological findings are coagulative necrosis, hepatocyte death, bile stasis, bile duct proliferation and lymphoplasmacytic infiltrate in peri-portal zone^50^. These histopathological aspects were observed 6 months after pristane injection in liver of Lgals3^−/−^ mice. This time was not sufficient to induce liver damages on Lgals3^+/+^ mice. At least in part, we suggested a critical associated between mesenteric disorganization with hepatic injuries in our experimental model. In accordance, a recent work revealing that deletion of mesenteric adipose tissue aggravated nonalcoholic fatty liver disease in mice^51^ reinforced our propose.

SLE is an autoimmune disease with etiology poorly understood, although a relationship between genetic components and environment is increasingly evident^52^. In this work, we proposed that Gal-3 controls autoimmune responses organizing B cell niches and fibrotic sites in the mesentery. For the first time, it is suggested a crosstalk between Gal-3, macrophage polarization, Notch pathways and DC differentiation in mesenteric structures associated with autoimmune hepatitis in lupus-like syndrome induced by pristane. Further studies will be valuable to correlate these findings in the mesentery with the pathogenesis of SLE.

## Supplementary information

Supplementary figures
