# Supplementary material for: Galectin-3 orchestrates the histology of mesentery and protects liver during lupus-like syndrome induced by pristane
Source: Sci Rep. 2019 Oct 10;9:14620. doi: 10.1038/s41598-019-50564-8 (PMC6786989; doi:10.1038/s41598-019-50564-8)
Supplement: Supplementary file 1 — Supplementary figures [file 41598_2019_50564_MOESM1_ESM.pdf]

Galectin-3 orchestrates the histology of mesentery and protects liver during lupus-like syndrome induced by pristane

Lemos FS[1]; Pereira JX[2]; Carvalho VF[3]; Bernardes ES[4]; Chammas R[5]; Pereira, TM[6]; Carvalho, RS[6]; Luisetto R[7]; El-Cheikh MC[8]; Calil-Elias S[1]; Oliveira FL[1,8]

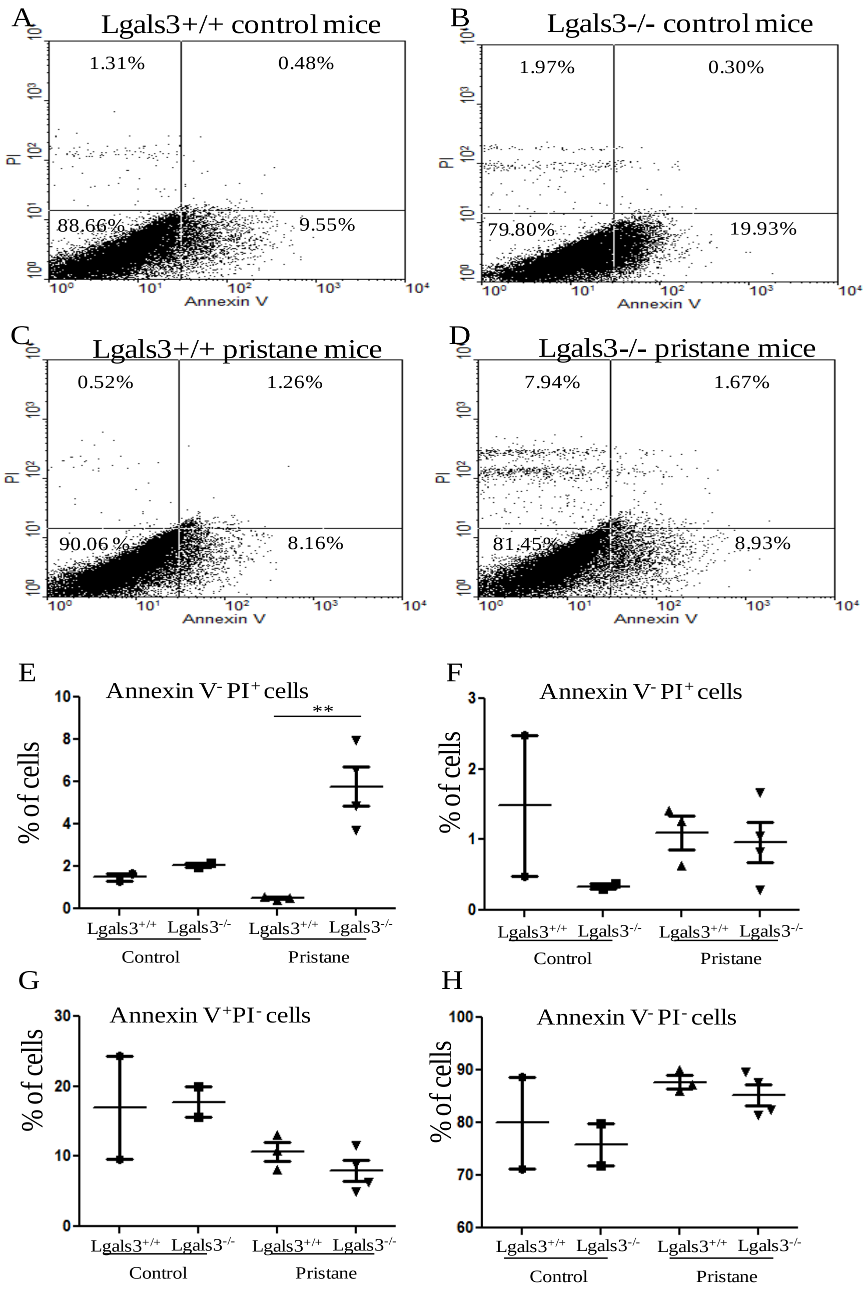

Supplementary Figure 1: Cell death in the mesenteric tissues submitted to pristane treatment for 6 months. Mesenteric dissociated cells were submitted to annexin-V and propidium iodide staining. (A-D) Representative dot plot graphs indicating the phenotype associated to Annexin V and PI in mesenteric cells of control Lgals3+/+ mice (A), control Lgals3-/- mice (B), pristane-induced Lgals3+/+ mice (C) and pristane-induced Lgals3-/- mice (D). The percentage of each population was showed in subsequent graphs indicating Annexin-V+PI+ cells (E), Annexin-V-PI+ cells (F), Annexin-V+PI- cells (G), and Annexin-V-PI- viable cells (H). Data are indicative of three independent experiments. ** shows statistical differences with P<0.01.

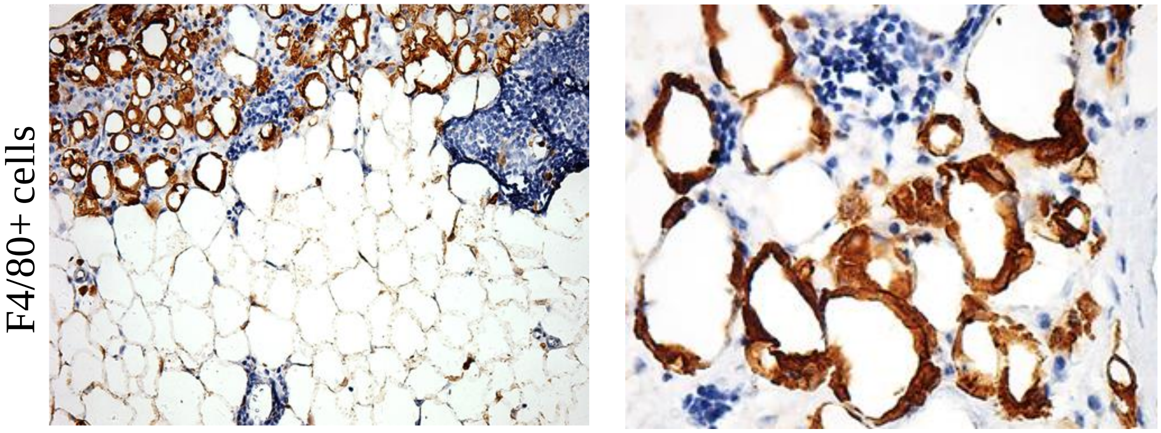

Supplementary Figure 2: Immunohistochemistry to F4/80 in mesenteric tissues of pristane-induced Lgals3$^{+/+}$ mice. (A) Cells expressing F4/80 were preferentially located in submesothelium and almost totally absent within lymphoid clusters. (B) High resolution of F4/80 staining surrounding crown-like structures. Magnification: A, 200x. B, 500x. These data are representative of three independent experiments.

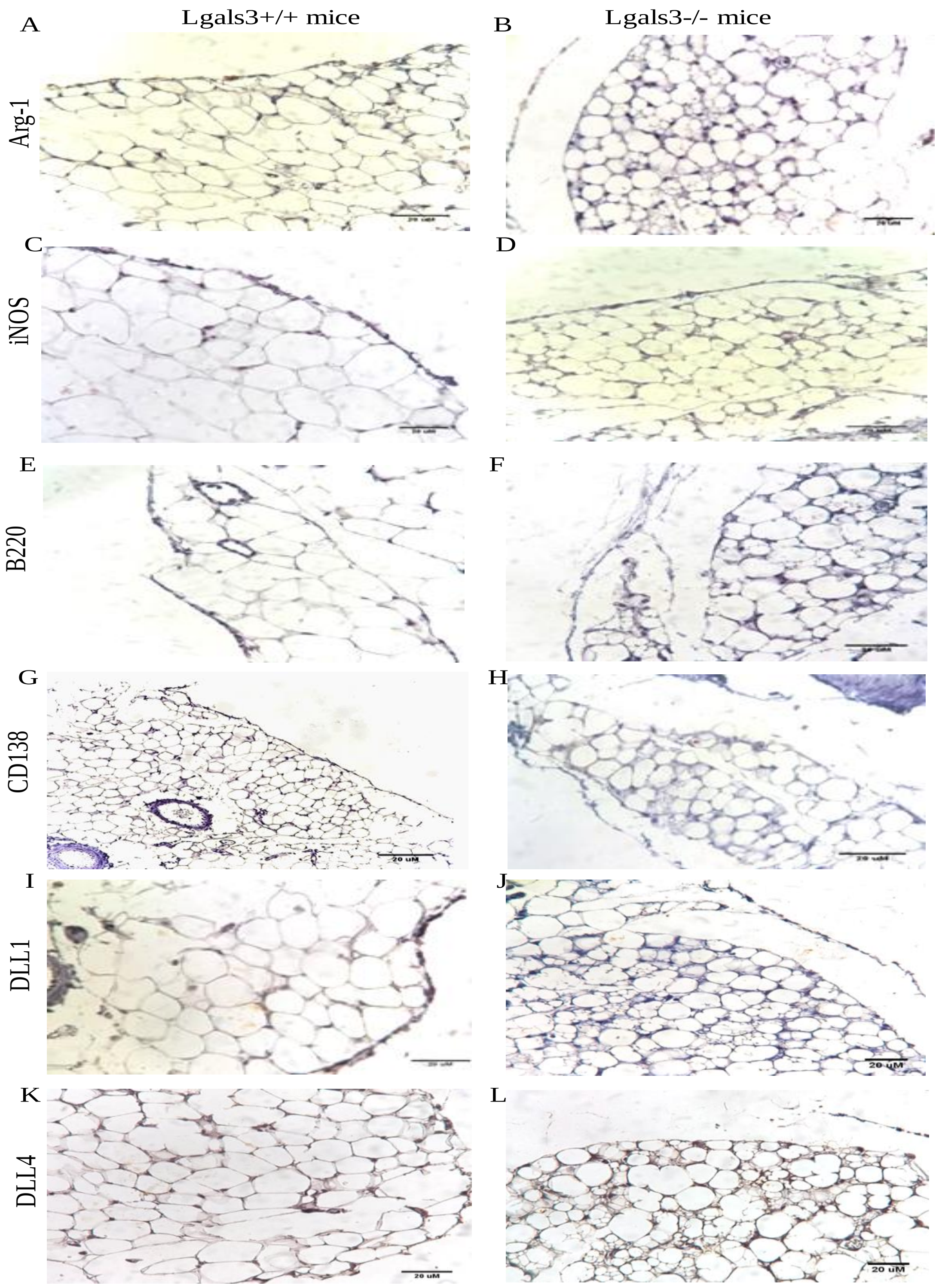

Supplementary Figure 3: Immunohistochemistry to mesenteric tissues of non-treat mice. Mesentery of Lgals3$^{+/+}$ and Lgals3$^{-/-}$ mice, both non stimulated with pristane, was stained to Arginase-1 (A and B, respectively), iNOS (C and D, respectively), B220 (E and F, respectively), CD138 (G and H, respectively), DLL1 (I and J, respectively), DLL4 (K and L, respectively). These data are representative of three independent experiments.
